# Improvement in Light Output of Ultraviolet Light-Emitting Diodes with Patterned Double-Layer ITO by Laser Direct Writing

**DOI:** 10.3390/nano9020203

**Published:** 2019-02-04

**Authors:** Jie Zhao, Xinghuo Ding, Jiahao Miao, Jinfeng Hu, Hui Wan, Shengjun Zhou

**Affiliations:** 1 Key Laboratory of Hydraulic Machinery Transients (Wuhan University), Ministry of Education, Wuhan 430072, China; zjie1994@whu.edu.cn (J.Z.); xinghuo@whu.edu.cn (X.D.); miaojh@whu.edu.cn (J.M.); 2017282080101@whu.edu.cn (J.H.); wanhui_hb@whu.edu.cn (H.W.); 2 Center for Photonics and Semiconductors, School of Power and Mechanical Engineering, Wuhan University, Wuhan 430072, China; 3 State Key Laboratory of Applied Optics, Changchun Institute of Optics, Fine Mechanics and Physics, Chinese Academy of Sciences, Changchun 130033, China

**Keywords:** UV LEDs, double-layer ITO, pinhole pattern, current spreading, light output power

## Abstract

A patterned double-layer indium-tin oxide (ITO), including the first unpatterned ITO layer serving as current spreading and the second patterned ITO layer serving as light extracting, was applied to obtain uniform current spreading and high light extraction efficiency (LEE) of GaN-based ultraviolet (UV) light-emitting diodes (LEDs). Periodic pinhole patterns were formed on the second ITO layer by laser direct writing to increase the LEE of UV LED. Effects of interval of pinhole patterns on optical and electrical properties of UV LED with patterned double-layer ITO were studied by numerical simulations and experimental investigations. Due to scattering out of waveguided light trapped inside the GaN film, LEE of UV LED with patterned double-layer ITO was improved as compared to UV LED with planar double-layer ITO. As interval of pinhole patterns decreased, the light output power (LOP) of UV LED with patterned double-layer ITO increased. In addition, UV LED with patterned double-layer ITO exhibited a slight degradation of current spreading as compared to the UV LED with a planar double-layer ITO. The forward voltage of UV LED with patterned double-layer ITO increased as the interval of pinhole patterns decreased.

## 1. Introduction

Ultraviolet (UV) light-emitting diodes (LEDs) have attracted considerable attention due to their potential applications in sterilization, water purification, photocatalyst, and solid-state lighting [1,2,3,4,5,6,7,8]. The improvement of light output is crucial for the realization of high-efficiency UV LEDs. The uniform current spreading is strongly correlated with light output and device reliability [9,10,11,12]. Thus, p-type electrodes possessing low contact resistivity as well as high transmittance have been extensively studied. Several kinds of transparent conductive layer such as metal nanowires [13,14,15,16], graphene [17,18,19], carbon nanotubes [20,21], and conductive polymers [22,23,24] have been reported to improve the current spreading of LEDs. For top-emitting LEDs, indium-tin-oxide (ITO) has been widely used to increase current spreading uniformity over the entire active region due to its high optical transmittance and excellent electrical conductivity [25,26,27]. In addition, thermal annealing process has been used to improve ohmic contact performance between ITO and p-GaN [28,29]. However, the ITO exhibits optical degradation at UV wavelength region during the thermal annealing process [30]. 

On the other hand, the light extraction efficiency (LEE) is limited by total internal reflection (TIR) because of large difference in refractive index between sapphire substrate, GaN film, ITO and the air. To overcome TIR at the ITO-air interface, microstructures formed on ITO have been reported to redirect the orientation of photons to favor emission into the air, leading to increasing scattering probability of photons at the ITO-air interface [31,32,33,34,35,36,37]. Moreover, compared to randomly distributed ITO pattern, the escape probability of photons emitting from the active region can be further increased by a regularly distributed ITO pattern [38]. However, patterned ITO has been shown to degrade the current spreading performance of UV LED due to an increase in sheet resistance [39,40,41]. Adding an annealed bottom ITO layer is an effective way to stabilize the current spreading and prevent electrical degradation. 

In this study, we combined two single ITO layers into double-layer ITO to obtain excellent ohmic contact and high LEE. The first ITO layer was annealed to form low-resistance ohmic contact with p-GaN. The unannealed second ITO layer was patterned to increase the scattering probability of photons at the ITO-air interface. The periodic pinhole patterns with different intervals were fabricated on the unannealed second ITO layer using laser direct writing. Effects of interval of pinhole patterns on the optical and electrical characteristics of UV LED with patterned double-layer ITO were studied in detail using numerical simulations and experimental investigations. 

## 2. Materials and Methods

**Figure 1:**
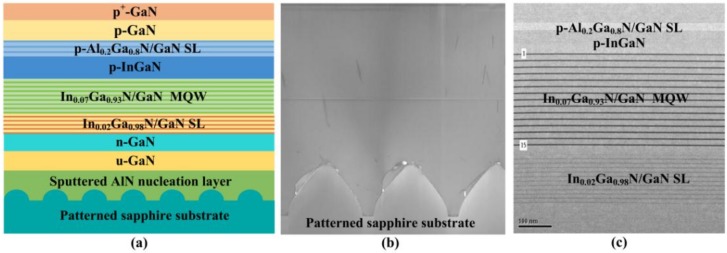
(**a**) Schematic representation of the UV LED epitaxial structure; (**b**) cross-sectional TEM image of the UV LED epitaxial structure; (**c**) magnified cross-sectional TEM image showing In_0.02_Ga_0.98_N/GaN SL, In_0.07_Ga_0.93_N/GaN MQW, and p-Al_0.2_Ga_0.8_N /GaN SL.

The GaN-based UV LED was grown on patterned sapphire substrate (PSS) with a sputtered AlN nucleation layer (15 nm) using the metal-organic chemical vapor deposition (MOCVD) technique. Trimethylgallium (TMGa), trimethylindium (TMIn), trimethylaluminum (TMAl), and ammonia (NH_3_) were used as precursors of Ga, In, Al, and N, respectively. Silane (SiH_4_) and bis (cyclopentadienyl) magnesium (Cp_2_Mg) were used as the n- and p-dopant sources, respectively. The epitaxial structure of UV LED includes a 15-nm-thick sputtered AlN nucleation layer, a 2.5-μm-thick undoped GaN layer, a 2-μm-thick n-GaN layer, a 12-pair In_0.02_Ga_0.98_N (2.1 nm)/GaN (2.3 nm) superlattice layer (SL), a 15-pair In_0.07_Ga_0.93_N (3 nm)/GaN (12 nm) multiple quantum well (MQW) active layer, a 28-nm-thick p-InGaN layer, a 5-pair p-Al_0.2_Ga_0.8_N (1 nm)/GaN(2 nm) SL, a 50-nm-thick p-GaN layer, and a 10-nm-thick p^+^-GaN layer. The peak emission wavelength of UV LED is 375 nm. Figure 1a shows a schematic representation of the 375 nm UV LED epitaxial structure. Figure 1b shows the cross-sectional transmission electron microscope (TEM) image of the UV LED epitaxial structure grown on PSS. Figure 1c shows the magnified cross-sectional TEM image of In_0.02_Ga_0.98_N/GaN SL, In_0.07_Ga_0.93_N/GaN MQW, p-InGaN layer, and p-Al_0.2_Ga_0.8_N /GaN SL. 

**Figure 2:**
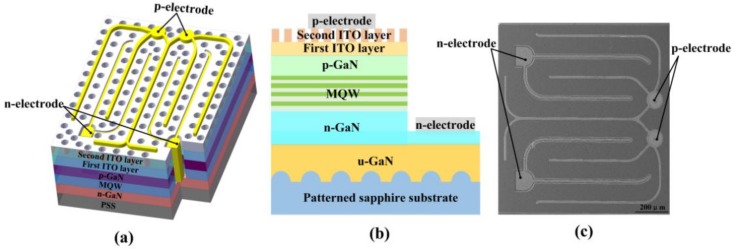
(**a**) Schematic representation of the UV LED with a patterned double-layer ITO; (**b**) cross-sectional schematic representation of the UV LED with a patterned double-layer ITO; (**c**) top-view SEM image of the fabricated UV LED chip.

UV LEDs with a patterned double-layer ITO were fabricated. The detailed fabrication process is composed of the following steps. Firstly, inductively coupled plasma (ICP) etching based on BCl_3_/Cl_2_ mixture gas was used to form GaN mesa by removing a portion of p-GaN layer and MQW active layer to expose the n-GaN layer (Step (1)). Then, the first 30-nm-thick ITO layer was subsequently deposited on the p-GaN layer using an electron beam evaporator, followed by thermal annealing in N_2_ ambient at 540 °C for 20 min to obtain good ohmic contact with p-GaN (Step (2)). Next, the second 120-nm-thick ITO layer was deposited on the first ITO layer, and the periodic pinhole patterns were then formed on the second ITO layer by laser direct writing (Step (3)). Finger-like Cr/Pt/Au (30 nm/30 nm/2000 nm) multilayers were adopted for both n-electrode and p-electrodes. The Cr/Pt/Au multilayers were deposited on the second patterned ITO layer for the formation of p-electrode. Meanwhile, the same Cr/Pt/Au multilayers were also deposited on the exposed n-GaN layer for the formation of the n-electrode (Step (4)). Finally, the UV LED wafers were thinned down to about 120 μm and diced into chips with dimension of 1132 × 1132 μm^2^ (Step (5)). Except for different interval of pinhole patterns fabricated on the second ITO layer, the fabrication process of these UV LEDs is identical. The diameters of pinhole patterns were 2 μm in UV LED II, UV LED III, and UV LED IV, while intervals of pinhole patterns were 400 nm, 600 nm, and 800 nm, respectively. For comparison, the UV LED I with planar double-layer ITO was also prepared. The light output power (LOP)-current-voltage (L-I-V) characteristics of UV LEDs were measured using an integrating sphere and a semiconductor parameter analyzer (Keysight B2901A). Figure 2a shows a schematic representation of the UV LED with a patterned double-layer ITO. Figure 2b shows a cross-sectional schematic representation of the UV LED with a patterned double-layer ITO. Figure 2c shows the top-view scanning electron microscopy (SEM) image of the fabricated UV LED chip. 

**Figure 3:**
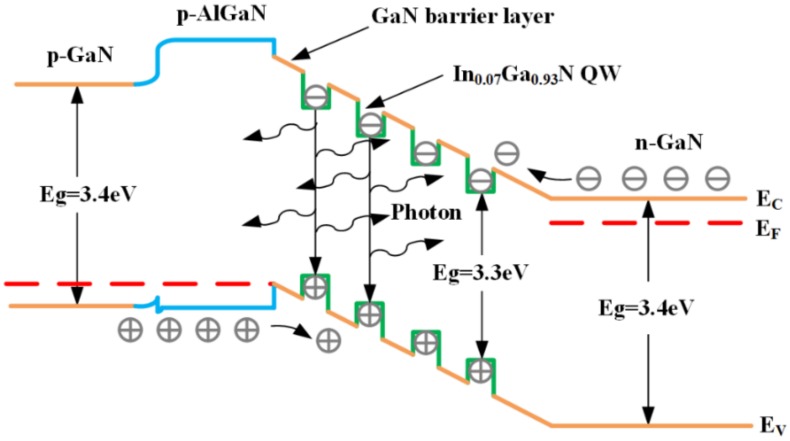
Energy band diagram of the UV LED at forward bias.

Figure 3 shows an energy band diagram of the UV LED at forward bias. When a current is passed through the UV LED, electrons and holes are injected into the In_0.07_Ga_0.93_N/GaN MQW active layer from the n-type GaN and p-type GaN layers, respectively. The electrons and holes recombine radiatively in the In_0.07_Ga_0.93_N/GaN MQW active layer, thereby emitting photons of light, as shown in Figure 3.

## 3. Results and Discussion

**Figure 4:**
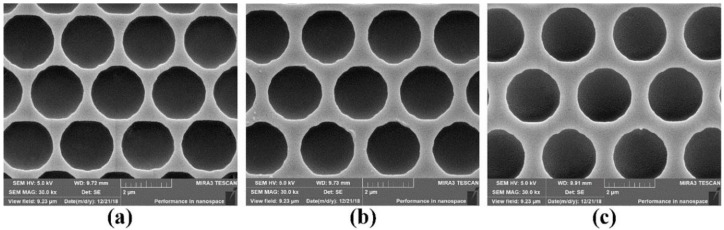
Top-view SEM images of the pinhole patterns with different intervals: (**a**) 400 nm; (**b**) 600 nm; (**c**) 800 nm.

The pinhole patterns on the second ITO layer were fabricated by laser direct writing system (4PICO.BV, Netherlands). First, the photoresist (ma-p1275HV) was spin-coated on GaN-based wafer at 4000 rpm for 60 s. Then, laser direct writing was performed on the photoresist film to obtain pinhole patterns, which will be transferred to the ITO film. The irradiating red laser beam (*λ* = 635 nm) was focused onto the surface of spin-coated photoresist through a dedicated objective lens (numerical aperture (NA) = 0.85, focal length = 0.6 mm, and magnification = 100×) to correct for surface errors. Then an ultraviolet laser beam (*λ* = 405 nm) was normally directed to the surface of photoresist with a spot diameter of 300 nm and an energy density of 60 mJ/cm^2^ through the same objective lens. The laser beam was scanned on the photoresist with a scan speed of 200 mm/s and stepping distance of 150 nm by a motorized stage. The wafer was then submersed into developer (ma-D 331) for 70 s to remove the area that has been exposed. Finally, pinhole patterns were transferred from photoresist onto the second ITO layer by wet chemical etching process. Figure 4 shows top-view SEM images of the pinhole patterns with different intervals. The diameter and intervals of the pinhole patterns of UV LED II, UV LED III, and UV LED IV were 2 μm/400 nm, 2 μm/600 nm, and 2 μm/800 nm, respectively. 

**Figure 5:**
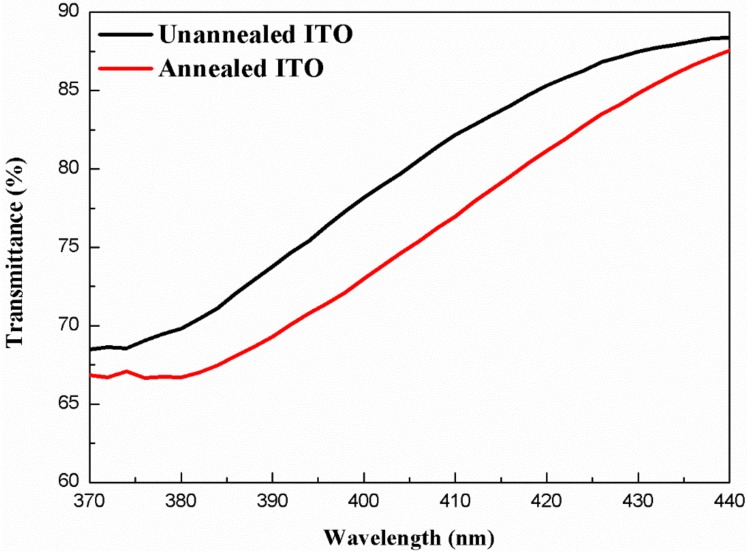
Transmission spectra of the unannealed ITO and annealed ITO films in the wavelength range of 370–440 nm.

We measured transmittance spectra of the evaporated ITO films before and after thermal annealing. Figure 5 shows the transmittance spectra of unannealed ITO and annealed ITO films in the wavelength range of 370–440 nm measured by a UV/VIS/NIR spectrometer. The transmittance of the unannealed ITO and annealed ITO were 68.81% and 66.88% at 375 nm, respectively. The transmittance of the unannealed ITO was 2.89% higher than that of the annealed ITO. It was indicated that the transmittance of ITO exhibited optical degradation at UV wavelength region after thermal annealing, which would lead to a decrease in LEE of UV LED. To avoid the decrease in transmittance of ITO at the UV wavelength region, the second ITO layer remains unannealed in our design. In addition, a combined method including electron beam evaporation and subsequent magnetron sputtering can further improve the optical transmittance of ITO [42]. 

**Figure 6:**
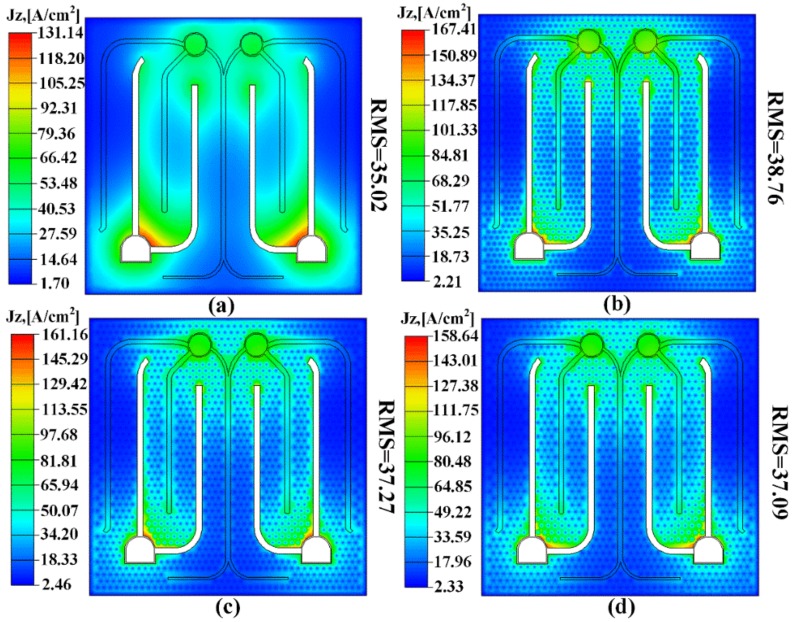
SimuLED simulation of the current density distribution in the active region of UV LED at 350 mA: (**a**) UV LED I; (**b**) UV LED II; (**c**) UV LED III; (**d**) UV LED IV.

We investigated the effect of interval of pinhole patterns on current spreading of UV LED with double-layer ITO using SimuLED commercial software package. Figure 6 shows simulation results of the current density distribution of UV LED I, UV LED II, UV LED III, and UV LED IV at 350 mA. The root mean square (RMS) of current density in the active region of UV LED I, UV LED II, UV LED III, and UV LED IV was 35.02 A/cm^2^, 38.76 A/cm^2^, 37.27 A/cm^2^, and 37.09 A/cm^2^, respectively. Compared with UV LED I, the RMS of current density in the active region of UV LED with patterned double-layer ITO was slightly increased. Meanwhile, the RMS of current density in the active region of UV LEDs increased with decreasing interval of the pinhole patterns on the second ITO layer. The slightly increased RMS of current density resulted from non-uniform current spreading.

**Figure 7:**
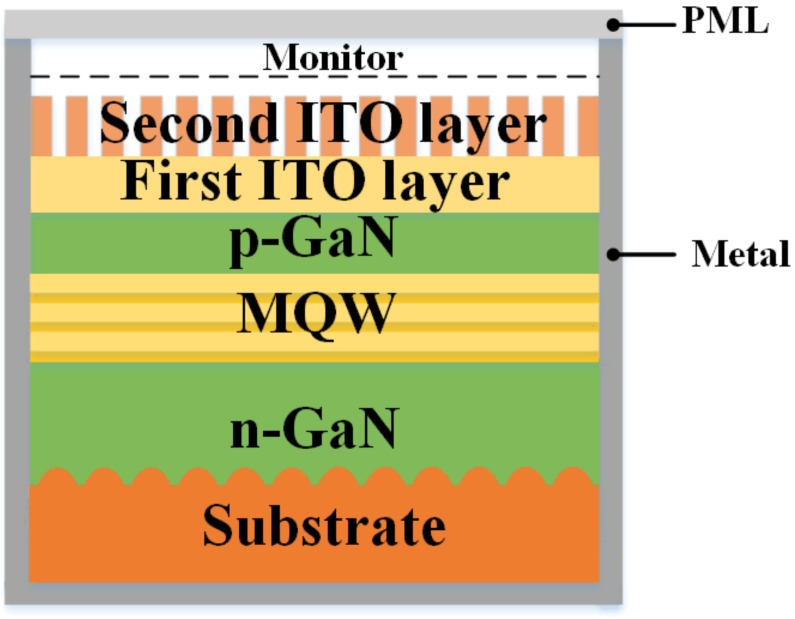
Schematic illustration of the FDTD simulation model for UV LED with a patterned double layer ITO.

The Finite-Difference Time-Domain (FDTD) method was applied for numerical simulation to clarify the effect of interval of pinhole patterns on the LEE of UV LED. To save computational memory and simulation time, the thickness of the sapphire substrate was fixed at 1 µm. Other structure parameters in the simulation were similar with the actual LED structure. The simplified UV LED structure model was depicted in Figure 7. The boundary conditions were a perfectly matched layer (PML) on top of the simulation region. The PML boundary absorbs incident light with zero reflection. Reflective metal boundaries were used at the bottom and four broad sidewalls. The top monitor was located 800 nm above the surface of the second patterned ITO to receive the optical waves extracted to air and calculate the LEE of the UV LED. 

In order to present the random propagation of unpolarized photons within the active region, sufficient dipole sources with wavelength of 375 nm were regularly distributed throughout the middle of the MQW layer with an interval of 1 μm. Three orientations of dipole sources were along the *x*-, *y*-, and *z*-axis. Compared to UV LED I, the LEEs of UV LED II, UV LED III, and UV LED IV were improved by 13.06%, 12.04%, and 7.02%, respectively. The enhancement in LEE was attributed to the formation of pinhole patterns on the second ITO layer, which redirected the photon propagation into the air escape cone and provided the photons with multiple opportunities to escape from the LED surface. 

**Figure 8:**
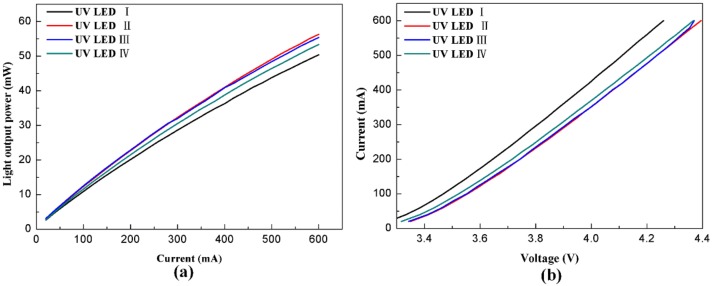
(**a**) L-I characteristic and (**b**) I–V characteristic of UV LED I, UV LED II, UV LED III, and UV LED IV.

The LOPs of UV LEDs versus injection current were shown in Figure 8a. At an injection current of 350 mA, the LOPs of UV LED I, UV LED II, UV LED III, and UV LED IV were 32.60 mW, 36.70 mW, 36.38 mW, and 34.75 mW, respectively. Compared with UV LED I, the LOPs of UV LED II, UV LED III, and UV LED IV were improved by 12.4%, 11.7%, and 6.5%, respectively. The improvement of LOP was attributed to increased LEE caused by formation of a pinhole pattern on the second ITO layer, as evidenced by FDTD simulation. Moreover, LOP of UV LED with a patterned double-layer ITO increased with the decrease of interval of pinhole patterns, owing to the higher density of pinhole pattern on the second ITO layer leading to an increased escape probability of photons. Figure 8b shows the I–V characteristic of UV LEDs. At 350 mA, the forward voltages of UV LED I, UV LED II, UV LED III, and UV LED IV were 3.88 V, 4.01 V, 3.99 V, and 3.97 V, respectively. The forward voltage of UV LED with patterned double-layer ITO was slightly higher than that of UV LED with planar double-layer ITO. The slightly higher forward voltage originated from the pinhole patterns on the second ITO that impeded lateral current spreading. Additionally, the forward voltage of UV LED with a patterned double-layer ITO increased with decreasing interval of the pinhole patterns. 

## 4. Conclusions

The first unpatterned current spreading layer and the second patterned light extracting layer were combined into a patterned double-layer ITO to improve current spreading and LEE of UV LEDs. The interval of the pinhole patterns on the second ITO has a significant impact on the sheet resistance of patterned double-layer ITO and the way of light propagation at the ITO-air interface. Compared to UV LED with planar double-layer ITO, LEE of UV LED with a patterned double-layer ITO was improved due to the scattering out of the waveguided light trapped inside the GaN film. The LOP of UV LED with a patterned double-layer ITO increased with decreasing interval of the pinhole patterns due to the higher density of pinhole pattern formed on the second ITO. Owing to non-uniform current spreading caused by pinhole patterns on the second ITO, the forward voltage of UV LED with a patterned double-layer ITO was slightly increased.
